# Anti-Inflammatory Effects of Curcumin-Based Nanoparticles Containing α-Linolenic Acid in a Model of Psoriasis In Vitro

**DOI:** 10.3390/nu17040692

**Published:** 2025-02-14

**Authors:** Simona Serini, Sonia Trombino, Roberta Cassano, Mariapaola Marino, Gabriella Calviello

**Affiliations:** 1 Department of Translational Medicine and Surgery, Section of General Pathology, School of Medicine and Surgery, Università Cattolica del Sacro Cuore, Largo F. Vito, 00168 Rome, Italy; mariapaola.marino@unicatt.it; 2 Fondazione Policlinico Universitario A. Gemelli IRCCS, Largo F. Vito, 00168 Rome, Italy; 3 Department of Pharmacy, Health and Nutritional Sciences, University of Calabria, Arcavacata di Rende, 87036 Cosenza, Italy; sonia.trombino@unical.it (S.T.); roberta.cassano@unical.it (R.C.)

**Keywords:** linolenic acid, curcumin, solid lipid nanoparticle, psoriasis, ferroptosis

## Abstract

**Background/Objectives.** Psoriasis is a common chronic skin inflammatory disorder pathogenetically associated with genetic, environmental, and immunological factors. The hallmarks of psoriatic lesions include sustained inflammation related to alterations in the innate and adaptive immune response, uncontrolled keratinocyte proliferation, differentiation, and death, as well as dysregulated crosstalk between immune cells and keratinocytes. In search of novel therapeutic strategies based on the use of natural products and dietary components to combine to the available conventional and innovative therapeutics, we explored the anti-inflammatory, antioxidant, and immunomodulatory activities of Curcumin (CU)-based solid lipid nanoparticles (SLNs) carrying the omega-3 fatty acid linolenic acid (LNA) in an in vitro model of psoriasis that had been previously constructed and characterized by us. **Methods.** This in vitro model consists of differentiated in vitro THP-1 macrophages (Mφs) and NCTC-2544 keratinocytes exposed or not to conditioned medium (CM) from Mφs treated with the Toll-like receptor-7 ligand imiquimod (IMQ). **Results.** In Mφs, the treatment with CU-LNA-SLNs inhibited the IMQ-induced expression of proinflammatory cytokines (IL-23, IL-8, IL-6: 43%, 26.5% and 73.7% inhibition, respectively, vs IMQ-treated Mφs), as well as the hyperproliferative response (12.8% inhibition vs IMQ-treated Mφs) and the increase in cell death observed in keratinocytes treated with Mφ-derived CM (64.7% inhibition). Moreover, in the same conditions, CU-LNA-SLNs reverted to control levels of the increased keratinocyte expression of two markers of ferroptosis, a form of death recently involved in the pathogenesis of psoriasis (TFRC and MDA: 13.4% and 56.1% inhibition, respectively). **Conclusions.** These results suggest that CU-LNA-SLNs could inhibit psoriatic inflammation, as well as the hyperproliferation and death of keratinocytes in psoriatic lesions, and could be considered as a new possible therapeutic strategy for psoriasis to be further evaluated for the topic treatment of psoriatic skin in vivo.

## 1. Introduction

Psoriasis is a chronic inflammatory disorder that mostly affects the skin [1]. It represents a quite common pathology of the skin, showing a worldwide prevalence of 2% [2,3]. The pathogenesis of this disease has been associated with genetic, environmental, and immunological factors, often related to each other’s, similarly to what is observed for other chronic inflammatory diseases [4]. Moreover, the uncontrolled proliferation of the keratinocytes and the alterations in their differentiation are considered further hallmarks of the psoriatic lesions [5], and later, they support the perpetuation of chronic inflammation through the production and secretion of inflammatory cytokines and chemokines [6,7,8,9].

Interestingly, the dysregulated crosstalk between immune cells and keratinocytes has also been recognized as a major step in the induction of psoriatic skin [10]. Particularly, we have been interested in the role played by the abnormal interactions between macrophages and keratinocytes in the pathogenesis of psoriasis. Our interest was related to the features of the skin macrophages that have the capacity to shift their phenotype in response to the changed condition of the microenvironment [11]. Due to this attitude, these cells can play opposite roles in the pathogenesis of psoriasis, either triggering psoriatic inflammation or exerting an anti-inflammatory activity. These properties have been considered optimal candidates for new therapeutic approaches [11].

It is also necessary to mention the role exerted in the pathogenesis of psoriasis by keratinocyte death and the consequent disruption of the epidermal barrier. However, the exact mechanism of their death is still under debate [12,13]. It has been found that psoriatic keratinocytes are able to better resist apoptosis [12] yet they are more susceptible to necroptosis [13] and, through the activation of the inflammasome, trigger inflammation [12,14]. Recently, however, a hypothesis has been put forward according to which ferroptosis, an iron-dependent non-apoptotic form of death, may have a relevant pathogenic role in the development of psoriasis [15,16,17]. In fact, increased oxidative stress occurs, with the following accumulation of lipid peroxidation end-products in the skin and circulation that is a peculiar characteristic of psoriasis [18]. It is known that ferroptosis occurs when dysregulation of the thiol-based redox system and increased oxidative stress occur in tissues [19]. It has also been reported that the increased levels of oxidative stress markers in psoriatic patients are associated with the duration and severity of the disease [18].

Currently, the therapy of psoriasis is based on three main approaches: topical therapy, systemic therapy, and phototherapy (for a review, see [20]). The topic therapy, particularly indicated for the treatment of mild and moderate psoriasis, is based on the use of a calcineurin inhibitor [21], a vitamin D3 analogue [22], and keratolytic agents [23], even though corticosteroids still represent the main first-line therapeutic approach [24]. The systemic approach is mainly used for the treatment of the most severe forms of psoriasis and is based on immunomodulating drugs, including retinoids and methotrexate [25], or on the use of different biologic drugs (including the anti-IL-17a secukinumab [26], the anti-IL-12 and anti-IL-23 ustekinumab [27], or the anti-TNF-α infliximab [28]), representing an effective alternative. However, they are very expensive and may become less efficient if administered for a long period to the patients [4,29,30]. The phototherapy, performed with psoralen + UVB, broad-band UVB, narrowband UVB [31], or excimer laser [32], is preferred for the therapy of psoriasis affecting large body surface areas. In any case, the available therapeutics share the feature to be able to improve symptomatology but not to eradicate the disease [33].

In search of safer treatments to substitute the existing ones or complement their action for a better result, there is growing interest in exploring the potential of bioactive natural compounds with known anti-inflammatory and antioxidant activities [33]. A very recent review article [20] focused on the phytochemicals studied so far for the treatment of psoriasis. Among these, aloe vera, a plant widely used for skin pathologies, contains a polysaccharide in gel form, which, besides possessing moisturizing activity, may exert a therapeutical action in psoriasis by inhibiting inflammation [34]. The plant alkaloid berberine is also able to exert an effective anti-inflammatory activity by reducing the expression of pro-inflammatory markers (such as COX-2, iNOS, TNF-α, and IL-12) in LPS-stimulated macrophages [35]. Moreover, recently, this phytochemical has been found to ameliorate skin lesions in mice with IMQ-induced psoriasis by suppressing the JAK1/STAT1 signaling pathway [36]. Furthermore, other plant extracts may be useful for the treatment of psoriasis due to their antiproliferative [37], moisturizing, and emollient properties [38]. Additionally, some of them may effectively reduce psoriatic itching, pain, and scaling [39,40].

Particularly, in the present work, we have focused on the *n*-3 polyunsaturated fatty acid (PUFA) α-linolenic acid (LNA, 18.3n:3) and curcumin (CU), which are plant-derived natural products that possess a very safe profile and are known for their antioxidant and anti-inflammatory effects [41,42]. We have been studying the health effects of the *n*-3 PUFAs in several neoplastic and inflammatory conditions for over thirty years [43,44,45,46]. Previous works, including ours [46], have demonstrated that *n*-3 PUFAs are able to modulate immune response in different inflammatory conditions of skin, including psoriasis [47], and they have been used as adjuvants in the therapy of a variety of cutaneous pathologies [48]. To this end, it is particularly interesting to underline that it was observed recently that lower levels of LNA were present in the plasma of psoriatic patients compared to healthy controls [49]. 

LNA is the metabolic precursor of the two long-chain *n*-3 PUFAs eicosapentaenoic (EPA, 20:5n-3) and docosahexaenoic acid (DHA, 22:6n-3), largely known for their health effects. However, we have chosen to investigate the potential of LNA in psoriasis based on our previous observation. In fact, we previously demonstrated that this essential fatty acid showed improved activity, thus representing a suitable alternative to EPA or DHA, when incapsulated in nanoformulations that were able both to deliver it efficiently to specific sites, as well as to protect it from any possible oxidative attack [45,50]. This is a very important point, considering that the use of LNA, due to its abundance in nuts and seed oils, would be much more sustainable than that of EPA or DHA, obtained mainly from marine fish. Moreover, it was previously observed that LNA has peculiar role in the skin, by participating in the maintenance of the barrier function, by inducing the terminal differentiation of keratinocytes and the formation of the stratum corneum, as well as in collaborating in the maintenance of an anti-inflammatory phenotype in that district by inhibiting the production of pro-inflammatory eicosanoids from arachidonic acid and pro-inflammatory cytokines [51]. Nonetheless, even though LNA, as well as DHA and EPA, is known to effectively contrast inflammation in vitro [52,53,54], it has been largely demonstrated that LNA is not physiologically or efficiently accumulated in human tissues [55]. Moreover, it was shown that after LNA, EPA, or DHA dietary supplementation, the levels of EPA and DHA markedly changed in plasma, while those of LNA remained almost unchanged [55]. In fact, it has been observed that a large amount of dietary LNA is oxidized, and also due to the limited interconversion of n-3 fatty acids in humans, LNA supplementation does not result in a significant accumulation of omega-3 PUFAs in plasma [55,56]. 

As far as CU is concerned, it is the main bioactive component present in *Curcuma longa*, a plant largely known for its numerous pharmacological activities [57,58], possessing antineoplastic and anti-inflammatory properties [59,60,61]. Particularly, CU was found able to inhibit the production of pro-inflammatory cytokines, including TNF-α, IL-17, IL-1β, and IL-6, and to exert remarkable antioxidant activities [62,63]. The safety and the efficacy of CU in the treatment of psoriasis were also demonstrated [64,65,66], even though it is also characterized by a very scarce bioavailability when administered in the native form [67]. For this reason, we aimed to overcome these drawbacks by including CU in the structure of solid lipid nanoparticles (SNPs) and to analyze the potential increased beneficial effects of LNA included in the CU-based LNA-containing SLNs.

In particular, here, we sought to explore the anti-inflammatory, antioxidant, and immunomodulatory activities of CU-based SLNs carrying LNA in an in vitro model of psoriasis consisting of differentiated in vitro THP-1 macrophages (Mφs) and NCTC 2544 keratinocytes. The use of CU-based SLNs was aimed to ensure an optimal intradermal penetration of the two bioactive natural compounds due to lipophilic properties of the SLNs, as well as to their capacity to protect the oxidation-prone LNA until its release in the damaged tissues. These SLNs were constructed and characterized previously by us [68]. In that study, we found that CU and LNA exerted remarkable antioxidant and anti-inflammatory activity when tested in an in vitro model of atopic dermatitis. For this reason, we have now analyzed them for their potential effectiveness against psoriasis.

In the present study, we evaluated the effect of the CU-LNA-SLNs on Mφs treated with the Toll-like receptor (TLR)-7 ligand imiquimod (IMQ). This compound is known to be a powerful immunomodulator and has been previously used to activate the pro-inflammatory activity of macrophages in models of psoriasis in vitro and in vivo [69,70]. The keratinocytes, treated or not with the SLNs, were then exposed to the conditioned medium (CM) obtained from IMQ-treated Mφs, to mimic the microenvironment of psoriatic skin and investigate the potential of the CU-LNA-SLNs in modulating the relationships that exist between keratinocytes and macrophages. Moreover, we have also investigated the effect of the SLNs on some factors involved in the induction of ferroptosis in keratinocytes exposed to CM obtained from IMQ-treated Mφs.

## 2. Materials and Methods

### 2.1. Cell Lines and Treatments

The human immortalized NCTC 2544 keratinocytes were kindly gifted by Dr. R. De bellis (Urbino University, Urbino, Italy) and were maintained in DMEM cell culture medium containing 2 mM glutamine and antibiotics (100 U/mL penicillin, 100 µg/mL streptomycin) (purchased from Sigma-Aldrich, St. Louis, MI, USA) in the presence of 10% fetal bovine serum (FBS). The THP-1 human monocytic cell line was obtained by the American Type Culture Collection (ATCC, Manassas, VA, USA) and was maintained in an RPMI 1640 cell culture medium containing glutamine (2 mM) and FBS (10%) (DMEM, RPMI and FBS were purchased from Biowest, Bradenton, FL, USA).

Both the cell lines were cultured at 37 °C in a humidified atmosphere at 5% CO_2_. Cells were maintained in the exponential growth phase by seeding them twice a week at a concentration of 3 × 10^5^ cell/mL.

Imiquimod (IMQ) (purchased from Sigma-Aldrich, St. Louis, MI, USA) was used to simulate the macrophage activation during the inflammatory process associated with psoriasis development. IMQ was dissolved in DMSO at the concentration of 2 mg/mL (corresponding to an 8.3 mM stock solution). From the stock solution, aliquots of 1.2, 3.6, and 6 µL/mL were used to obtain the final IMQ concentrations of 10, 30, and 50 µM in cell culture plates.

The lyophilized samples of empty curcumin-based solid lipid nanoparticles (CU-SLN) or containing linolenic acid (CU-LNA-SLN) were dissolved in cell culture medium at the concentration of 2 mg/mL. From the stock solutions, different aliquots were used in order to obtain the final concentrations of 0.5, 1, and 5 µg/mL. For the preparation of the SLNs, pure Curcumin and Linolenic acid were obtained from Sigma-Aldrich (St. Louis, MI, USA). The exact procedure used for the synthesis of the SLNs, as well as their physical-chemical properties, has been previously reported [70].

### 2.2. MTT Cell Viability Assay

The MTT assay was used to evaluate the metabolic activity of cells as an indicator of cell viability. This colorimetric method is based on the reduction in the 3-(4,5-dimethylthiazol-2-yl)-2,5-diphenyltetrazolium bromide (MTT), a yellow-colored compound, to blue-colored formazan crystals by the metabolically active cells. Viable cells contain the mitochondrial enzyme succinate dehydrogenase, which transforms the tetrazolium ring of MTT into formazan. The insoluble formazan crystals are dissolved by DMSO and then the absorbance of the blue-colored solution is measured by a plate spectrophotometer at a 570 nm wavelength.

The NCTC 2544 and THP-1 cells were seeded at the concentration of 5 × 10^3^ cells/well in a 96-well multiwell cell culture plate in a final volume of 200 µL. The THP-1 cells were first treated with phorbol myristate acetate (PMA, 320 nM) to induce their differentiation into Mφs. The differentiation was demonstrated by both the change in the morphology of cells and by the observation that, differently from the undifferentiated THP-1 cells, the differentiated macrophages adhere to the bottom of the wells. After 24 h, the cell culture medium was removed and substituted with fresh cell culture medium containing the empty CU-SLN or the CU-LNA-SLN at the concentrations of 0.5, 1, and 5 µg/mL. At the indicated time points (24, 48, and 72 h), 50 µL of MTT solution (2 mg/mL in PBS) were added to each well, and the cell culture plates were further incubated for 4 h at 37 °C. Then, the supernatant was removed, and the formazan crystals were solubilized with DMSO (100 µL/well). Absorbance was measured at 570 nm and at 630 nm (the basal absorbance to be subtracted to the 570 nm absorbance) by using a plate spectrophotometer (Tecan, Männedorf, Switzerland). Cell viability was calculated by using the following formula:

% Viable cells= (Absorbance ^(570-630)^ treated cells/Absorbance ^(570-630)^ control cells) ×100

### 2.3. Trypan Blue Cytotoxicity Assay 

Through this experiment, we wanted to evaluate the possible cytotoxic effect of the CM derived from the THP-1 cells, differentiated and treated with IMQ, in the absence or in the presence of the CU-LNA-SLN on the NCTC 2544 keratinocytes. Cells were seeded at the concentration of 3 × 10^5^ cells/well in a 60 mm Petri dish. After 24 h, cell culture medium was removed and substituted with CM obtained from the macrophages treated for 24 h with 50 µM IMQ and 5 µg/mL CU-LNA-SLNs, alone and in combination. After further 24 h, cells were trypsinized, centrifuged, and counted by a Neubauer chamber.

### 2.4. Real-Time Quantitative PCR Analysis of IL-23, IL-8, IL-6, IL-6R, and TFRC Expression

Total RNA was isolated from cell suspensions using the RNeasy Mini Kit (Qiagen GmbH, Hilden, Germany) following the manufacturer’s instruction and analyzed through the NanoDrop Microvolume Spectrophotometer (Thermo Fisher Scientific, Waltham, MA, USA). Retrotrascription was performed by using the Omniscript RT kit (Qiagen, Germantown, MD, USA) based on the following reaction: 2 μL of 10 Buffer RT, 2 μL of 5 μM dNTPs Mix for each dNTP, 2 μL of 10 μM oligo-dT primer, 1 μL of 10 units/μL RNase inhibitor, 1 μL of inverse transcriptase (4U for each reaction), and 5 μL of RNA for a maximum concentration of 2 μg in a final reaction volume of 20 μL in RNase-free H_2_O. The reaction was performed in a thermal cycler (Eppendorf Mastercycler Gradient, Eppendorf, Milan, Italy) at 37 °C for 1 h. The obtained cDNA has been used as a template for the quantitative real time PCR (qRT-PCR). The qRT-PCR has been performed by using the SsoAdvanced Universal Probes Supermix and the PrimePCR Gene Expression Assays specific for IL-23, IL-8, IL-6, IL-6R, and TFRC (Bio-Rad Laboratories, Hercules, CA, USA). The RT-PCR has been performed with the CFX96^TM^ Real-Time System (Bio-Rad Laboratories). The expression of the target genes has been normalized to the level of the β2-microglobulin gene, as an internal control.

### 2.5. MDA Assay for the Evaluation of Lipid Peroxidation 

Malondialdehyde (MDA), the main product of lipid peroxidation, was evaluated by the Lipid peroxidation (MDA) Colorimetric/Fluorometric Assay Kit (APExBIO, Boston, MA, USA) following the manufacturer’s instructions. Briefly, 1 × 10^6^ cells (corresponding to 10 mg of proteins) for each sample were homogenized on ice with 300 µL of MDA Lysis Buffer containing 3 µL of BHT (100×). Samples were then centrifuged at 13,000× *g* for 10 min to remove the insoluble particulate matter. The supernatant (200 µL) was then placed in a clean microcentrifuge tube. In the meantime, an MDA standard curve was obtained by diluting 10 µL of the MDA contained in the kit in 407 µL of bidistilled water to prepare a 0.1 M MDA solution. Then, 20 µL of the 0.1 M solution were diluted with 980 µL of bidistilled water to obtain a 2 mM MDA solution. For the colorimetric analysis, 0, 2, 4, 6, 8, and 10 µL of the 2 mM MDA solution were diluted to a 200 µL final volume to obtain the final MDA concentrations of 0, 4, 8, 16, and 20 nM. In each supernatant sample, 600 µL of thiobarbituric acid (TBA) was then added. The samples were incubated at 95 °C for 60 min and then placed on ice for 10 min. The samples (200 µL) were placed in the wells of a multiwell 96-well culture plate, and the absorbance was measured at a 532 nm wavelength. The concentration of MDA in the samples was calculated by extrapolation from the MDA standard curve using the following formula:

C = [(A/mg)] × 4 × D = nmol/mg

where

A = MDA nmoles of samples extrapolated for standard curve;
mg = protein amount in the sample (in mg); 4 = correction factor (from the original reaction mix, only 200 µL were utilized);
D = potential dilution factor used before the analysis.

### 2.6. Statistical Analysis

The data were analyzed using the one-way analysis of variance (ANOVA) followed by Tukey’s test. The analysis was performed using the InStat GraphPad Software 10.1.2. (324) version (San Diego, CA, USA).

## 3. Results

In the present study, we used an in vitro cellular model mimicking the inflammatory process underlying the development of psoriasis, one of the most common skin diseases. To this aim, we used Imiquimod (IMQ), a Toll-like receptor (TLR)-7 ligand and powerful stimulator of macrophage activity [69], which was largely used to activate the macrophages pro-inflammatory activity in vitro [70]. Our in vitro model of psoriasis consisted of macrophages differentiated from THP monocytes (Mφs) by using 320 nM PMA and then exposed to IMQ. The CM obtained from the IMQ-treated Mφs were then added to NCTC 2544 keratinocyte cultures.

In order to validate this model of psoriasis in vitro in our experimental condition and establish a suitable and not cytotoxic IMQ concentration to be used in further experiments, we first treated TPH-derived macrophages and NCTC 2544 keratinocytes separately with increasing IMQ concentrations. The need to also perform such a preliminary experiment on NCTC 2544 cells was related to the fact that these cells would be subsequently exposed to the CM of IMQ-treated Mφs.

**Figure 1:**
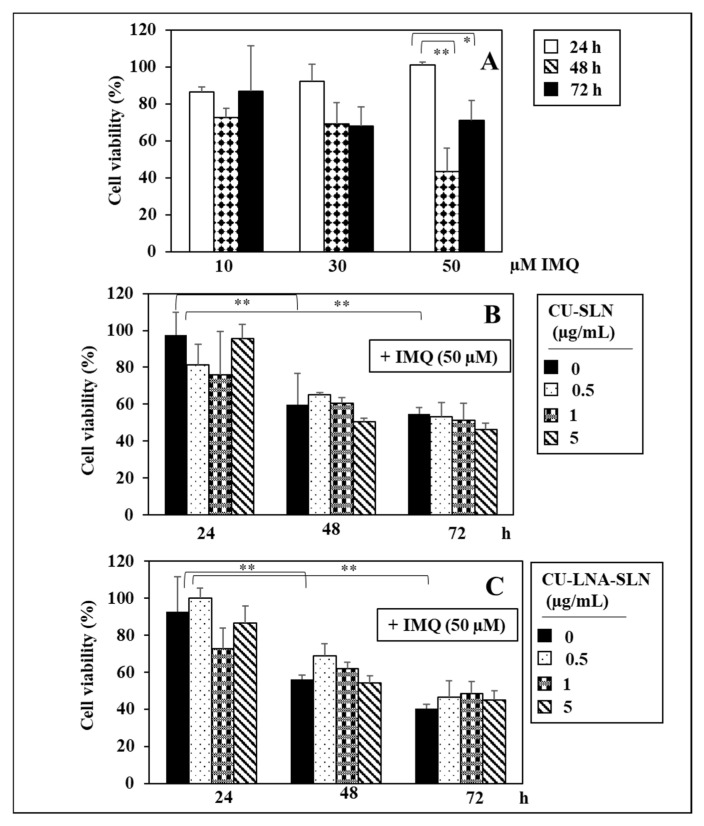
Effect of increasing doses of 10–50 µM IMQ in THP-1 differentiated cells (Mφs) treated for 24–72 h (**A**); Effect of increasing doses of CU-SLNs (**B**) and CU-LNA-SLNs (**C**) on Mφs treated with 50 µM IMQ for 24–72 h. Data represent the means ± SD of three different experiments. Values indicated with asterisks are significantly different from the control value [*: *p* < 0.05; **: *p* < 0.01, One-Way Analysis of Variance (ANOVA), followed by Tukey’s test].

Figure 1 shows the effect of increasing doses of IMQ (10–50 µM) in Mφs. We observed that, after 24 h treatment, none of the IMQ concentrations induced a significant reduction in cell viability (Figure 1A), which always remained in the range of 87–100%. Since MMT is used to evaluate the metabolic activity of cells and particularly the function of mitochondrial respiration, the large reduction in the values obtained through the MTT test at the high concentrations of IMQ suggests that doses higher than 10 µM may exert an inhibitory effect on the metabolic respiratory activity of Mφs, especially after a 3 day-treatment. For these reasons, we limited the concentrations of IMQ used in the further experiments performed on Mφs maximally to 50 µM IMQ for 24 h, to maintain the metabolic activity of these cells unaltered, in agreement with the physiological role that the TLR ligands are known to exert, i.e., triggering the pro-inflammatory activity of macrophages.

Thus, we studied the effect of the CU-based solid lipid nanoformulations (CU-SLNs containing or not containing LNA) on the Mφs treated for 24 h with 50 µM IMQ. Particularly, Figure 1B,C shows the effects of increasing concentrations of the empty CU-SLNs or of those containing LNA (CU-LNA-SLNs) on the metabolic activity of Mφs. It can be observed that neither the empty CU-SLNs nor the CU-LNA-SLNs were able to modify the viability of Mφs treated with IMQ 50 µM at any of the times analyzed, even though the CU-LNA-SLNs did not show a significant tendency to increase cell viability. In the other experiments, however, we decided to restrict the evaluations to periods of time comprised between the 6 h and the 24 h of treatment, when IMQ was not able to significantly alter the Mφ metabolic activity.

**Figure 2:**
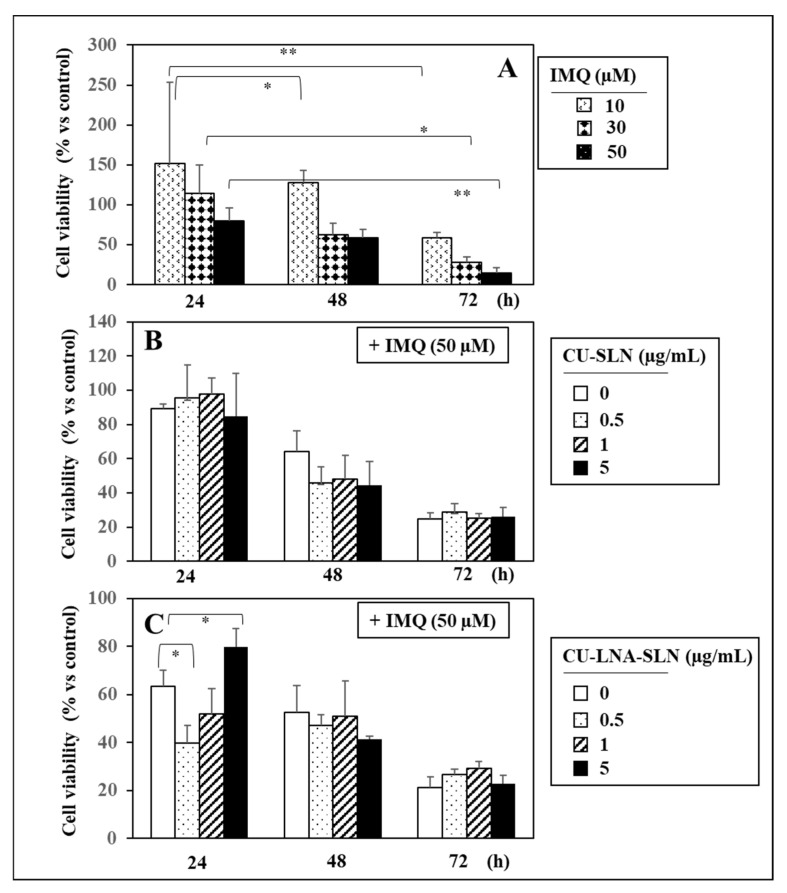
Effect of increasing doses of IMQ (10–50 µM) in NCTC 2544 keratinocytes treated for 24–72 h. (**A**); effect of increasing doses of CU-SLNs (**B**) and CU-LNA-SLNs (**C**) on NCTC 2544 keratinocytes treated with 50 µM IMQ for 24–72 h. Data represent the means ± SD of three different experiments. Values indicated by asterisks are significantly different from the control value [*: *p* < 0.05; **: *p* < 0.01, One-Way Analysis of Variance (ANOVA), followed by Tukey test].

Figure 2 shows the effects of increasing doses of IMQ on immortalized human NCTC 2544 keratinocytes, a cell line scarcely differentiated that represents an in vitro model for the cells of the basal layer of the epidermis. For this characteristic, they are often used to specifically investigate the involvement of the basal cells in studies focusing on the pathogenesis of skin chronic inflammatory disorders [71,72]. In this study, we have decided to use NCTC 2544 cells to verify whether the pro-inflammatory effects of IMQ, as well as the potential effects of the SLNs under study, could involve the cells belonging to the deeper epidermis layer.

In this case, no significant effect on cell viability was observed with the lowest dose of IMQ (10 µM), unless the treatment was prolonged until 72 h, when the cell viability was found suddenly and drastically halved. On the other hand, with the higher doses (30 and 50 µM), a time-dependent reduction in cell viability was observed that resulted substantially reduced after 72 h (by 73% and 86%, respectively) (Figure 2A). It is of note that empty CU-SLNs were never able to modify the reducing effect on cell viability induced by 50 µM IMQ, regardless of the CU-SLN concentrations analyzed (Figure 2B). On the contrary, after 24 h of treatment, the highest concentration used by us of CU-SLNs containing LNA (5 µM) was able to revert the cell viability values to levels approaching 80% of the control value. On these bases, for the remaining experiments, we decided to limit our evaluations to the effect of 5 µM CU-LNA-SLNs on cells treated or not with 50 µM IMQ.

**Figure 3:**
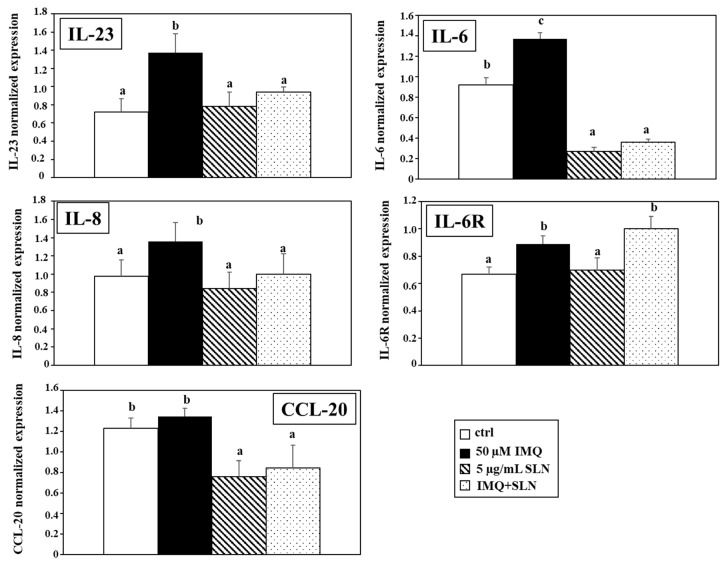
Effect of a 6 h-treatment with IMQ (50 µM) and CU-LNA-SLN (5 µg/mL), administered, alone and in combination, to Mφs on the expression of the pro-inflammatory cytokines IL-23, IL-6, and IL-8, of the chemokine CCL-20, and of the IL-6R, evaluated through qRT-PCR. Data are the means ± SD of a triplicate experiment. Within each panel, values not sharing the same superscript letters (a, b, and c) are significantly different [*p* < 0.05, one-way analysis of variance (ANOVA) followed by Tukey’s test].

Afterward, we decided to investigate the effect of IMQ on the Mφ expression of the IL-23, IL-6, and IL-8 cytokines, as well as that of the CCL-20 chemokine, known for being produced at a high level by macrophages in the psoriatic skin microenvironment and for being involved in the hyperproliferative response of the keratinocytes in psoriasis. Figure 3 shows the effects of 50 µM IMQ, both in the presence and in the absence of 5 µg/mL CU-LNA-SLN on the expression of the IL-23, IL-6, and IL-8 cytokines, as well as that of the IL-6R and CCL-20 chemokine, all evaluated through qRT-PCR. In fact, it is known that macrophages infiltrate extensively in the psoriatic derma in vivo, where they release a series of inflammatory cytokines [73,74,75,76]. Moreover, we studied the potential effect of a treatment with 5 µM CU-LNA-SLNs on the secretory activity of the Mφs. Finally, we evaluated the effect of IMQ and SLNs on the expression of the IL-6 receptor (IL-6R), with the aim of understanding whether the IL-6 production by Mφs could also exert an autocrine effect and amplify the pro-inflammatory activity.

We observed that the treatment with 50 µM IMQ induced a significant increase in the expression of IL-23, IL-6, and IL-8 as compared to the control condition (by 90%, 49%, and 39%, respectively, *p* < 0.05). On the other hand, in our experimental conditions, the expression of the chemokine CCL-20 was not significantly altered by the IMQ treatment.

In our experimental model, we observed that the treatment of Mφs with CU-LNA-SLNs alone did not significantly modify the IL-23 expression as compared to the control. On the other hand, when Mφs were exposed simultaneously to CU-LNA-SLNs and IMQ, the NPs were able to substantially inhibit the IL-23 overexpression induced by 50 µM IMQ (by 43%, *p* < 0.05), reverting the levels of this cytokine to values comparable to those observed in the control conditions.

Instead, we observed that the treatment of Mφs with 5 µg/mL CU-LNA-SLNs alone was sufficient to induce a dramatic decrease in IL-6 (70.7% inhibition, as compared to the control, *p* < 0.05), a cytokine found particularly increased in serum and skin lesions of patients with psoriasis [77,78]. Moreover, we observed that a comparable and even higher inhibitory activity was exerted by the SLNs when they were administered in concomitance to 50 µM IMQ (73.7% inhibition, as compared to the treatment with IMQ alone, *p* < 0.05).

However, we found that the CU-LNA-SLNs did not alter the Mφ expression of the IL-6 receptor, either in basal conditions or in the presence of 50 µM IMQ, suggesting that the nanoformulation was not able to reduce the potential autocrine effect of the IL-6. However, we found that that 50 µM IMQ was able by itself to induce the IL-6 receptor expression (33% induction, as compared to control cells, *p* < 0.05).

We then observed that a lack of effect of the CU-LNA-SLNs administered alone on macrophage IL-8 secretion corresponded a significant reduction in the increased production of this cytokine obtained when the CU-LNA-SLNs were given in combination with IMQ 50 µM (26.5% inhibition as compared to IMQ alone, *p* < 0.05). This effect was similar to that observed for IL-23, even though, in this case, it is of a more limited proportion (inhibition: 26.5% vs. 73.7%).

We also found that the treatment of the Mφs with CU-LNA-SLNs was able to induce a significant inhibition of the chemokine CCL-20 production, either administered alone or in combination with 50µM IMQ (38.2% and 37.2% reduction, as compared to control cells and cells treated with 50µM IMQ, respectively, *p* < 0.05).

**Figure 4:**
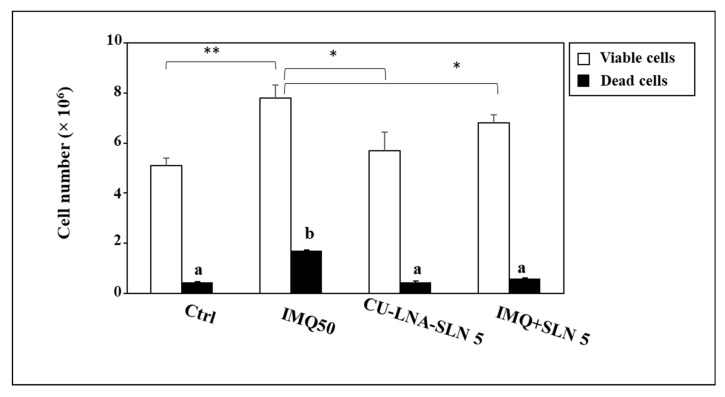
Effect of CM obtained by Mφs treated with 50 µM IMQ and CU-LNA-SLNs (5 µg/mL), alone and in combination, on the growth and viability of NCTC 2544 keratinocytes following a 24-h treatment. Data are the means ± SD of three different experiments. Values with asterisks indicate a significant difference (*: *p* < 0.05; **: *p* < 0.01); values not sharing the same superscript letters (a and b) are significantly different [one-way analysis of variance (ANOVA) followed by Tukey’s test].

Interestingly, it was previously observed that the cytokines produced by tissue macrophages play a crucial role in determining the hyperproliferation of keratinocytes observed in psoriatic lesions [79,80]. On these bases, we investigated the effect exerted on the proliferation of the NCTC 2544 keratinocytes by the CM obtained by Mφs cultured in the presence of IMQ alone or in combination with CU-LNA-SLNs (Figure 4). We observed that the CM obtained from Mφs exposed to IMQ (IMQ-CM) induced a significant increase in the number of keratinocytes after 24 h (measured by Trypan blue exclusion method, 53% increase as compared to control cell population, *p* < 0.05). On the other hand, the CM obtained by Mφs treated with CU-LNA-SLNs alone did not significantly modify the keratinocyte proliferation, as compared to control cells. Of note, however, the effect of the IMQ-CM obtained by Mφs exposed also to CU-LNA-SLNs significantly reduced the hyperproliferative effect of IMQ-CM, suggesting that this nanoformulation could have the potential to specifically target the abnormal proliferation of keratinocytes in the psoriatic lesions.

We also observed (Figure 4) that the CM obtained by Mφs exposed to IMQ induced a remarkable increase in the percentage of dead keratinocytes as compared to the CM obtained in control conditions (18% and 8.8% of the total number of cells, respectively). Conversely, the CM obtained by Mφs cultured in the other experimental conditions, i.e., treated with CU-LNA-SLNs alone and in combination with IMQ, did not alter the percentage of dead keratinocytes, as compared to the control (7.9% and 8.8%, respectively). Altogether, these findings suggest that CU-LNA-SLN could exert an indirect protective effect toward the keratinocyte population by acting primarily on the functions of the macrophages activated in a proinflammatory sense, present in the psoriatic area.

Furthermore, since ferroptosis has been reported to be a form of cellular death pathogenetically implicated in several inflammatory disorders, including psoriasis [16], we decided to investigate, in our in vitro model of psoriasis, whether CU-LNA-SLNs could be able to reduce the death of the keratinocytes induced by CM obtained from IMQ-treated Mφs, by negatively modulating ferroptosis and, in particular, some factors involved in this kind of death.

**Figure 5:**
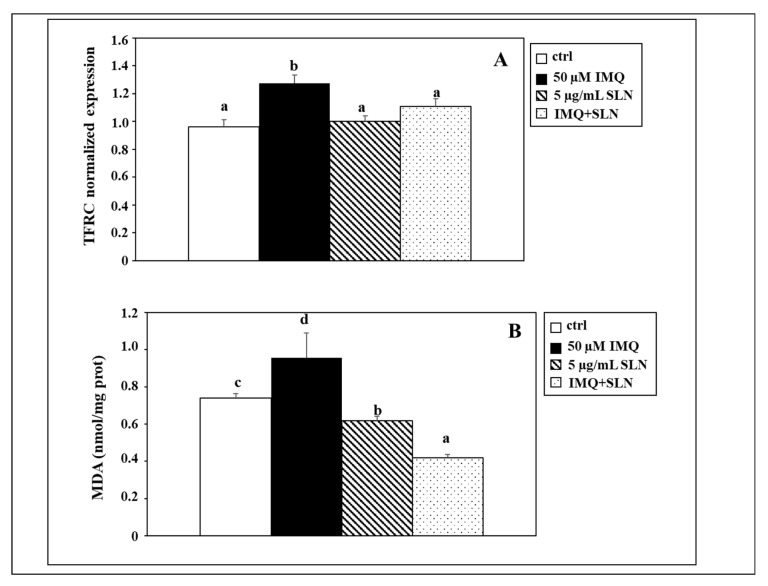
Effect of IMQ (50 µM) and CU-LNA-SLNs (5 µg/mL) on the expression of transferrin receptor (TFRC) (**A**) and on the MDA production (**B**) in NCTC 2544 keratinocytes in the presence of Mφ-derived CM. Data are the means ± SD of a triplicate experiment for TFRC and of three different experiments for MDA. Values not sharing the same superscript letters (a, b, c, and d) are significantly different [*p* < 0.05, one-way analysis of variance (ANOVA), followed by Tukey’s test].

On these bases, we exposed NCTC 2544 keratinocytes to the CM obtained by Mφs exposed to 50 µM IMQ (IMQ-CM), either in the absence or in the presence of 5.0 µg/mL CU-LNA-SLNs and evaluated the expression of the transferrin receptor (TFRC) by using qRT-PCR and, as a further marker of ferroptosis, the production of MDA, the main end-product of lipid peroxidation (Figure 5). We observed that the treatment with IMQ-CM induced an increase in the TFRC expression by keratinocytes (33% increase, as compared to control cells, *p* < 0.05), thus confirming the hypothesis that an increased ferroptosis is involved in the pathogenesis of psoriasis.

Moreover, we observed that, even though the CU-LNA-SLNs administered singularly did not significantly alter the TFRC levels, they were able to contrast the IMQ-CM -induced TFCR increase, allowing it to restore the levels of the control condition (13.3% inhibition vs. 50 µM IMQ, *p* < 0.05).

Accordingly, the IMQ-CM treatment induced a significant increase in the keratinocyte MDA production (31.5% increase, as compared to the control condition, *p* < 0.05). In this case, however, even CU-LNA-SLNs administered alone were able to inhibit MDA production and lipid peroxidation (31.5% increase, as compared to the control condition, *p* < 0.05), and the effect was much more marked when CU-LNA-SLNs were given along with IMQ-CM (56.2% inhibition, as compared to cell treated with 50 µM IMQ, *p* < 0.05). Such an effect is probably due to the high degree of antioxidant efficacy elicited by these NPs in basal conditions and observed previously by us in the phase of their chemical-physical characterization [68]. This effect, in addition to their ability to inhibit the TFRC expression reported now by us, could explain the capability that the SLNs have to contrast lipid peroxidation in keratinocytes, both in control conditions and in the presence of the prooxidant effect exerted by IMQ-CM.

## 4. Discussion

The data obtained in the present work on IMQ cytotoxicity confirmed what was previously obtained by Chuang et al. [70], who treated THP-differentiated cells for 24 h with increasing concentrations of IMQ (from 1.0 µg/mL to 20 µg/mL), even though it should be noted that the concentrations used by Chuang et al. [70] and corresponding to 0.2 µM to 4.0 µM IMQ were much lower than the ones used by us. However, it should be underlined that their experimental conditions were quite different since we used a solution of pure IMQ dissolved in DMSO for the treatment, allowing the identification of the specific pro-inflammatory activity of this compound. Chuang et al. [70] exposed the cells to a 5% IMQ cream formulation. This does not exclude the fact that other compounds contained in the same formulation could have influenced the IMQ activity. In any case, as we continued the IMQ treatment for a further 24 h (i.e., 48 h from the beginning of the experiment), we observed a contained but significant decrease in Mφ viability in the presence of the lowest IMQ doses (10 e 30 µM: 28% and 31% cell viability decrease, respectively). The decrease, however, became considerable in the presence of the highest dose (50 µM IMQ: 57% cell viability decrease).

The results obtained treating the NCTC 2544 keratinocytes with IMQ (Figure 2) confirm that an inductor of psoriasis such as IMQ acts also by directly damaging keratinocytes and validates the IMQ-induced in vitro model of psoriasis in our experimental conditions, where damage-associated molecular patterns (DAMPs) are released by damaged keratinocytes and induce the sustained inflammatory response typical of this disease.

In this study, we also evaluated the ability of the CU-LNA-SLNs to modulate the production of the inflammatory cytokines IL-23, IL-6, and IL-8 (Figure 3) since they are known to play crucial roles in the development of the psoriatic lesion [73,74,75,76]. Particularly, IL-23 has been identified as the cytokine mainly implicated in the proinflammatory activity of macrophages during the development of psoriasis [75], and the use of antibodies against IL-23 has recently been shown to be a highly efficient therapeutic strategy to rapidly reduce psoriatic lesions [81]. Moreover, macrophages also secrete the chemokine CCL20, reported to activate the specific class of lymphocytes Th17, known to underlie and sustain the psoriatic inflammatory response [82,83]. In this regard, it is interesting to notice that our in vitro model of psoriasis is based specifically on the capacity of IMQ to function as a selective antagonist for the Toll-like receptor-7 (TLR7) present on the surface of macrophages, lymphocytes, and dendritic cells. Thus, through the activation of the transcription factor NF-κB, IMQ stimulates these cells to produce a wide number of pro-inflammatory cytokines [84,85]. Our results suggest that the nanoformulation, administered at 5 µg/mL, besides being harmless to the Mφs and not able to modify the basal IL-23 cytokine secretion, may have the potential to modulate the overexpression of this cytokine observed in inflammatory conditions, such as those related to the development and progression of psoriatic lesion.

The massive decrease observed by us in the production of IL-6 by Mφs results is particularly interesting since this cytokine has been shown to be involved in the induction of the dysregulation of lipidic and glycemic metabolism developing in psoriatic patients as a consequence of chronic inflammation [86,87]. Moreover, it has been seen that the inhibition of this cytokine is more effective than any other treatment in decreasing the joint destruction in particular subpopulations of patients affected by psoriatic-related arthritis [88]. Thus, these findings suggest that the treatment with CU-LNA-SLNs, due to their ability to maintain extremely low the levels of IL-6, both in basal conditions and after the IMQ-induced pro-psoriatic inflammation, could represent not only a strategy to decrease the onset and progression of the pathological manifestations of psoriasis-related inflammation but also to prevent the dysmetabolic disorders, as well as the arthritic complications that can result from long term psoriasis.

Our finding that IMQ alone is able to induce an increased expression of the IL-6R is worth noticing, since, to the best of our knowledge, this is the first time that the IMQ-induced induction of this receptor is observed and suggests that it may represent an important mechanism in the amplification of the pro-inflammatory response typical of the psoriatic process.

Of particular interest was also the significant inhibition of the Mφ chemokine CCL-20 production obtained when the cells were treated with CU-LNA-SLNs, either administered alone or in combination with 50µM IMQ. In fact, chemotaxis represents a key factor in the crosstalk existing between immune cells and keratinocytes since the recruitment of immune cells in psoriatic lesions is strictly dependent on the presence/production of proteins showing chemotactic activity (such as chemokines and their receptors) in that area [89]. Particularly, CCL-20 represents a crucial factor in the infiltration of immunity cells into psoriatic lesions [90], being able to recruit dendritic cells and Th17 lymphocytes to sustain the inflammatory response through positive feedback mechanisms [91]. Accordingly, it has also been reported that the increased secretion of CCL-20 by macrophages induces the recruitment of monocytes in the psoriatic lesions of the mouse [92].

Our results (Figure 4) also showed that the treatment of NCTC 2544 keratinocytes with the IMQ-CM obtained by Mφs increased the proliferation of these cells, a feature resembling the hyperproliferation of keratinocytes in the psoriatic lesions [79,80], and that the treatment with the CU-LNA-SLNs was able to revert this effect. The presence of LNA in our CU-based nanoformulation may be relevant to this respect since, recently, Simard et al. [93] demonstrated that the treatment with LNA was able to reduce keratinocyte proliferation in a model of engineered skin tissues obtained by cells from healthy donors or psoriatic patients.

Ferroptosis has been reported to be a form of cellular death pathogenetically implicated in several inflammatory disorders, including psoriasis [16]. A variety of molecular factors have been implicated in the development of the ferroptosis process for their ability to regulate lipid peroxidation and the intracellular levels of iron [94]. Among these factors, the glutathione peroxidase 4 (GPX4) activity, as well as the levels of reduced glutathione (GSH) and cysteine in ferroptosis, have been largely studied [95]. Our particular attention, however, was drawn to TFRC, a factor recently found deeply implicated in the development of an uncontrolled lipid peroxidation inside the cells and considered a central marker of ferroptosis [96]. In fact, the increased level of this receptor, by enhancing the entrance of iron into the cells, may contribute to an enhancement of the Fenton reaction, thus favoring the ferroptotic form of cellular death.

## 5. Conclusions

Based on all the results obtained, we can conclude that in our model of psoriasis, in vitro, the treatment with CU-based-SLNs carrying the omega-3 PUFA LNA was able to substantially contrast the reduced metabolic activity, hyperproliferative reaction, and cell death of NCTC 2544 keratinocytes induced directly by IMQ or following the keratinocyte exposure to the CM obtained from IMQ-activated Mφs. IMQ induced Mφs to increase the expression of the IL-23, IL-6, and IL-8 cytokines. These results confirm the central trigger role played by the macrophages in the pathogenesis of psoriasis. Moreover, we have observed that the CU-LNA-SLNs were able to inhibit the IMQ-induced expression of IL-23 and IL-8 in Mφs, as well as their basal and IMQ-induced expression of IL-6, but not the expression of the IL-6 receptor, known to be involved in the IL-6-induced autocrine macrophagic response.

Furthermore, in agreement to the keratinocyte hyperproliferative response reported in psoriasis, we observed a significant increase in the number of keratinocytes exposed to CM obtained from IMQ-treated Mφs, and this increase in cell growth was accompanied by a concomitant rise in the number of dead cells. However, the addition of CU-LNA-SLNs to the NCTC-2544 cells treated with Mφ-derived CM was able to reduce both the total keratinocyte number as well as that of dead cells to the values observed in control conditions. This finding suggested that the CU-LNA-SLNs have the potential to improve these pathological manifestations typical of the psoriatic skin.

Finally, we suggested that ferroptosis could be one of the forms of death induced in keratinocytes by the CM derived from IMQ-treated Mφs since this treatment increased the expression/levels of two crucial markers of ferroptosis (i.e., TFRC and MDA) in keratinocytes. Of interest, the simultaneous addition of CU-LNA-SLNs to cultured keratinocytes exposed to Mφ-derived CM was able to revert the expression/levels of these markers of ferroptosis to values comparable to those found in control cells.

Overall, the results obtained suggest that CU-LNA-SLNs could have the potential to inhibit the development and progression of psoriatic inflammation, as well as the hyperproliferation and death of keratinocytes typical of psoriatic lesions. These properties could be considered as a new possible therapeutic strategy for psoriasis and be further evaluated for the topic treatment of psoriatic skin in vivo.

## Data Availability

The raw data supporting the conclusions of this article will be made available by the authors on reasonable request.
